# Dataset of quantitative proteomic analysis to understand aging processes in rabbit liver

**DOI:** 10.1016/j.dib.2020.105701

**Published:** 2020-05-15

**Authors:** Bushra Amin, Renã A.S. Robinson

**Affiliations:** a Department of Chemistry, Vanderbilt University, Nashville, TN 37235; b Department of Neurology, Vanderbilt University Medical Center, Nashville, TN 37232; c Vanderbilt Memory & Alzheimer's Center, Nashville, TN 37212; d Vanderbilt Institute of Chemical Biology, Nashville, TN 37232; e Vanderbilt Brain Institute, Nashville, TN 37232

**Keywords:** Aging, Rabbit, Liver, Proteomics, TMT

## Abstract

Here, we present a proteomics dataset of liver proteins to understand aging in rabbits, which complements the publication “Quantitative proteomics to study aging in rabbit liver” [1]. This dataset was generated to understand the molecular basis and metabolic changes of aging processes in liver, which is the main organ involved in metabolism, detoxification, transport, and signaling. Proteins from young, middle, and old age rabbits were extracted and digested. Generated peptides were labeled with light or heavy dimethyl groups at their N-termini, while lysine amines were labeled with TMT10-plex using a cPILOT workflow [2]. Labeled peptides were fractionated by basic pH reverse phase chromatography and analyzed with online reverse phase LC coupled with tandem mass spectrometry (MS/MS and MS^3^). The RAW files were generated using a Fusion Lumos Orbitrap mass spectrometer (Thermo Scientific) and processed with Proteome Discoverer (PD) version 2.2 to generate a list of identified and quantified proteins. Data was searched against the Rabbit UniProtKB redundant database. A total of 3,867 proteins were identified corresponding to 2,586 protein groups and 22,229 peptides. Dynamic levels of age-related proteins associated with fat metabolism, mitochondrial dysfunction, and protein degradation were detected. The mass spectrometry proteomics data (RAW files) and processed Proteome Discoverer 2.2 files (MSF files) have been deposited to the Proteomics Identification Database (PRIDE) ProteomeXchange Consortium and can be accessed with the dataset identifier PDX013220 (http://www.ebi.ac.uk/pride/archive/projects/PXD013220).

Specifications table

**Table:** 

Subject	Analytical Chemistry
Specific subject area	Aging, Proteomics, Metabolism
Type of data	Table Figure Mass spectra (RAW files) Proteome Discoverer (PD) readout files (MSF files)
How data were acquired	Liquid chromatography coupled to high-resolution mass spectrometer (LC – MS/MS). Quantification at MS^3^ level with cPILOT labeling strategy. LC parameters: Nano-flow UHPLC (Dionex) with auto sampler; 120 min gradient. MS parameters: Fusion Lumos Orbitrap MS (Thermo Scientific), Top speed selection (3 sec) data dependent acquisition (DDA) method. Top 10 synchronous precursors were selected for MS^3^ level quantification.
Data format	Raw mass spectrometry files, un-filtered PD read-out files, and analyzed PD excel files
Parameters for data collection	Male, New Zealand white rabbits were categorized as young (252 days), middle (683 days), and old age (1350 days) groups according to their age. Liver was dissected to extract proteins. Extracted proteins were digested with a mixture of trypsin and Lys-C proteases. Peptides were differentially labeled with cPILOT strategy (low pH dimethylation label at peptides N-termini and high pH TMT10-plex labeling at lysine amine). Labeled peptides were desalted, pooled, and used for proteomics analysis.
Description of data collection	[Four animals of similar age and sex were included in each group [biological variation (CV = 0.29)]. Three technical injections (CV= 0.27) and an internal standard that carried equimolar proteins from all animals have enabled us to quantify statistically significant proteins which were dynamic in the liver of old age rabbits*.* Offline basic pH reverse phase fractionation was used to reduce the complexity and increase the depth of the liver proteome in this 14-plex experiment.
Data source location	Nashville, Tennessee, United States of America (USA)
Data accessibility	Proteomics Identification Database (PRIDE) ProteomeXchange Consortium: Dataset identifier: PDX013220 http://www.ebi.ac.uk/pride/archive/projects/PXD013220
Related research article	Amin B, Robinson, RAS. Quantitative Proteomics to Study Aging in Rabbit Liver. Mechanisms of Ageing and Development, 187, 2020, 111227

## Value of the data

• This data provides quantitative and qualitative information about liver proteins which are specific to aging in rabbits.
• This data may be useful to understand the mechanism of aging as well as factors that lead to age-associated metabolic diseases.
• This data extends existing knowledge of the molecular mechanisms of aging processes in the liver*.*
• Rabbits are one of the least explored models to understand complex *in vivo* biological processes particularly with proteomics; this data will increase the known proteins and pathways for this species.
• This data supports rabbit as an attractive model of complex aging processes.

## 1 Data

**Fig. 1:**
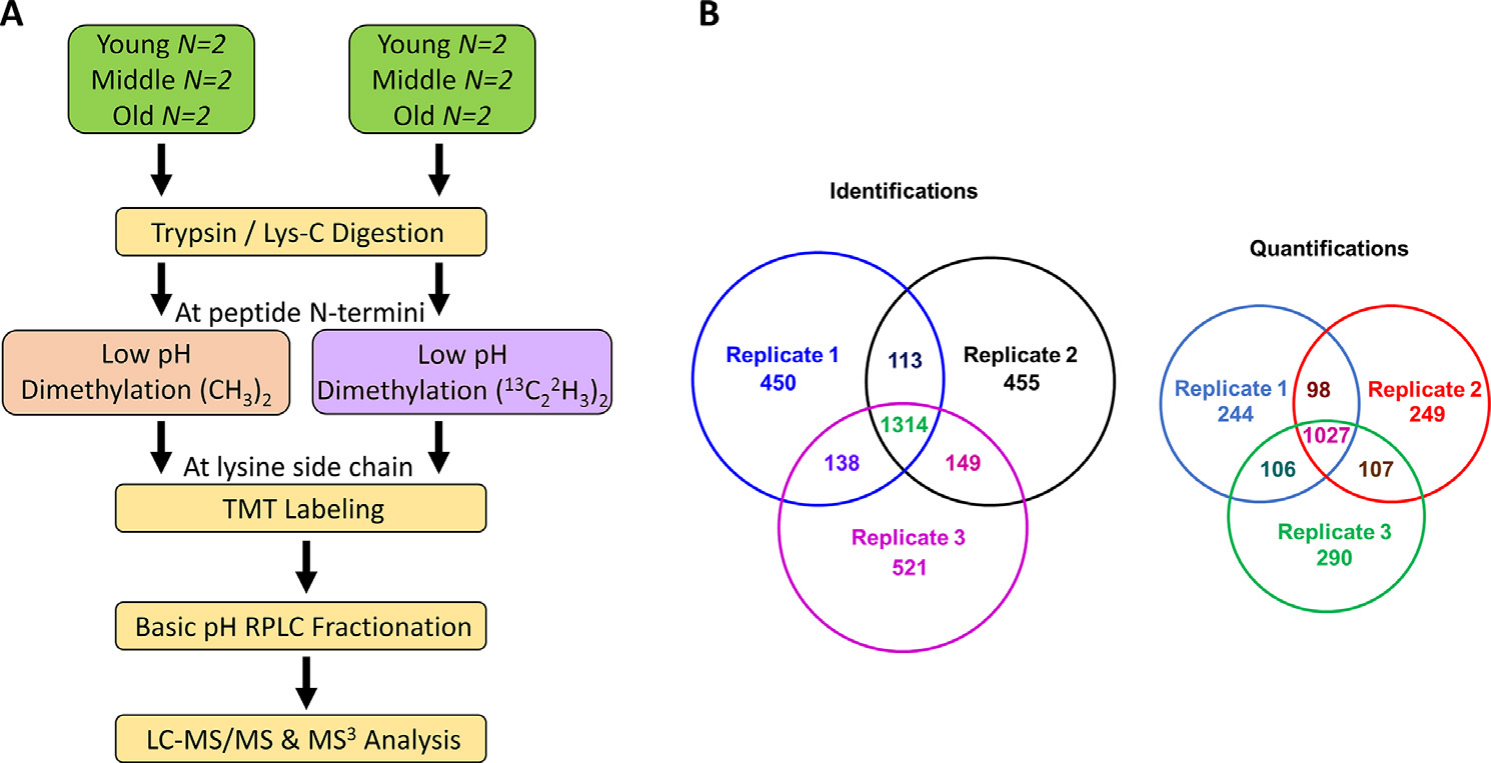
**Experimental workflow and number of identified and quantified proteins: A)** New Zealand white rabbits of mean age 252 days (N=4), 683 days (N=4), and 1350 days (N=4) were categorized as young, middle, and old age groups, respectively. Liver proteins were extracted and digested with a trypsin / Lys-C mixture. Peptides were labeled using the cPILOT strategy, where peptide N-termini were labeled with light or heavy dimethyl groups at pH < 3.0, while lysine amines were labeled with TMT10-plex labels at pH 8.5. cPILOT-labeled peptides were pooled and fractionated with basic pH reverse phase chromatography prior to their analysis on an Orbitrap Fusion Lumos MS. cPILOT-labeled peptides were fragmented with CID-MS/MS, and the 10 most intense MS/MS fragments were subjected to HCD fragmentation at the MS^3^ level for the detection of TMT reporter ions. **B)** Venn diagrams of the total number of identified and quantified proteins across the three technical replicates. Quantified proteins represent those identified proteins with S/N ratios above the threshold for all of the reporter ion channels.

**Fig. 2:**
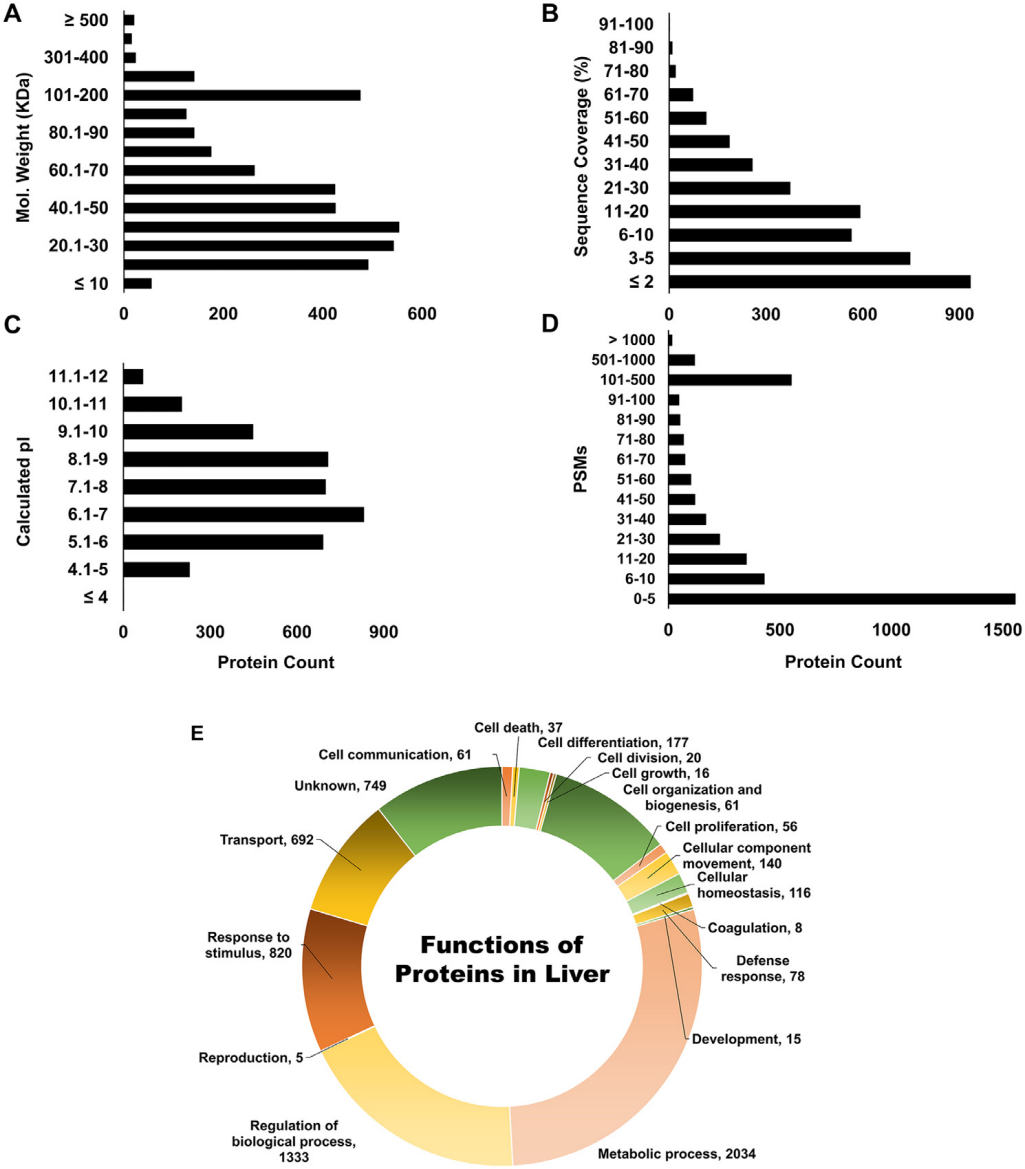
**Bar graphs of the properties of identified proteins in rabbit liver. A**) Labeled peptides from all biological replicates and internal standards were subjected to triplicate technical analysis on an Orbitrap Fusion Lumos MS. Greater than five hundred proteins were identified with a molecular weight ≤ 50 kDa, ∼ 200 proteins were identified with a molecular weight between 60-70 kDa, and ∼ 500 proteins were identified with higher molecular weight between 100-200 kDa. **B**) A majority of proteins had 20 % or higher sequence coverage, and **C**) calculated isoelectric points of identified proteins were between 4.0 to 12.0. **D**) A majority of proteins (i.e., ≥ 1500) were identified with up to 5 peptide spectral matches (PSMs), ≥ 400 proteins each were identified with 10 and 20 PSMs, while ≥ 950 proteins were identified with 30-100 PSMs. **E**) Donut graph with the functions (GO terms) of identified proteins (N=2586) in liver.

**Fig. 3:**
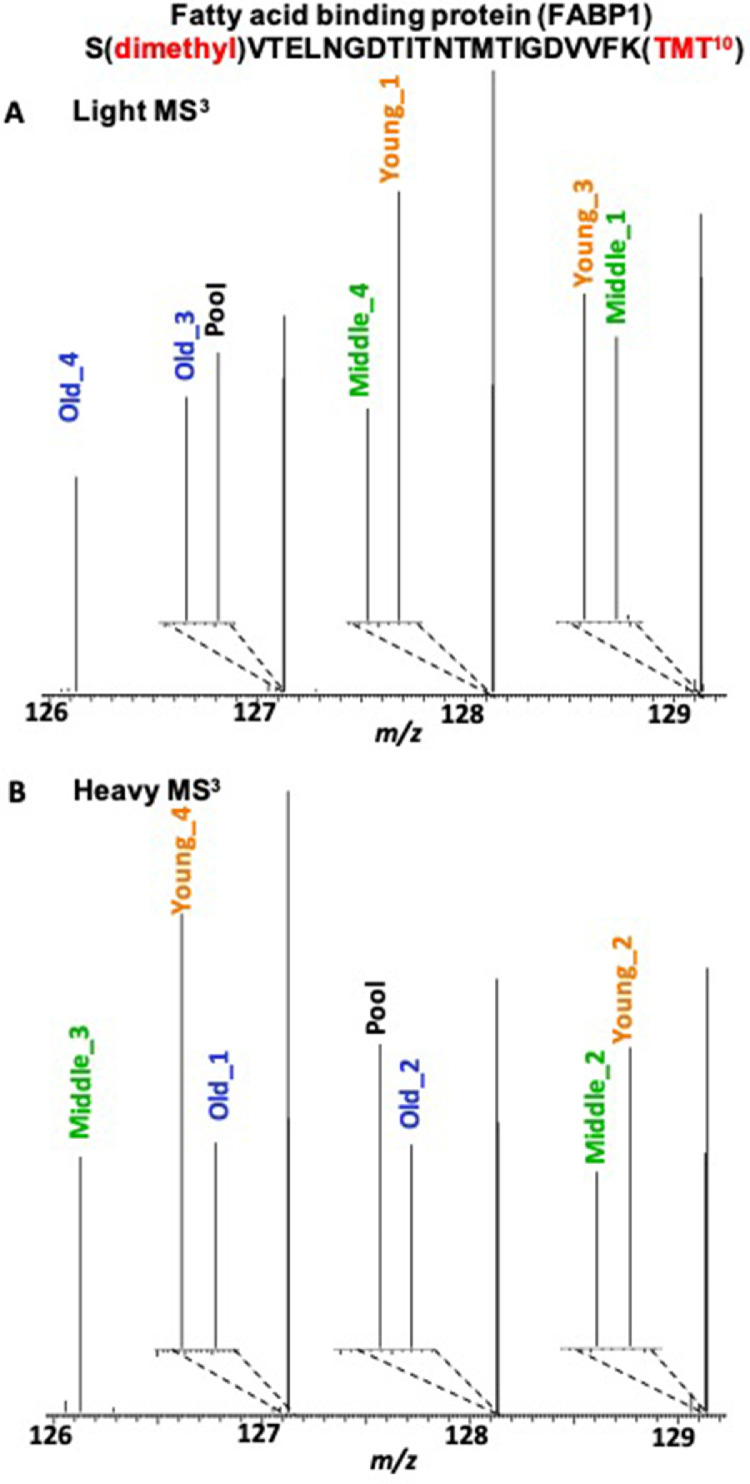
**Dynamic levels of fatty acid binding protein-1 (FABP1) across young, middle, and old age rabbits.** Labeled peptides were analyzed by LC-MS/MS and MS^3^ analysis on a Fusion Lumos Orbitrap mass spectrometer. TMT reporter ion intensities were detected at the MS^3^ level in the Orbitrap. Example MS^3^ spectra of FABP1 are shown for a **A)** light and **B)** heavy dimethylated peptide of FABP1. Lower levels of FABP1 were detected with increasing age.

Data presented here was generated by mass spectrometry-based proteomic analysis of rabbit liver proteins from young, middle, and old age rabbits [1]. Rabbits were divided into two groups to have equal representation of each age in both groups for light (CH_3_)_2_ and heavy (^13^C^2^H_3_)_2_ dimethyl labeling at peptide N-termini. An internal standard was generated by mixing equimolar amounts of all the samples. This dataset includes RAW and mass spectrometry read-out files (MSF). Generated RAW files were processed using Proteome Discoverer (PD) version 2.2 and searched against the Rabbit UniProtKB redundant database. Proteins and peptides were filtered to generate final read-out files. Table 1 provides biological and technical variation of this dataset with or without normalization. Fig. 1A displays the experimental workflow used to prepare and analyze samples. Fig. 1B depicts the number of proteins identified and quantified across three technical runs. Fig. 2 demonstrates the molecular weight (**2A**), sequence coverage (**2B**), isoelectric point (**2C**), peptide-to-spectral matches (PSMs) of identified proteins (**2D**), and functions of identified proteins (**2E**). Fig. 3 gives example MS^3^ spectra for a peptide from fatty acid binding protein-1 with reporter ion signals detected in young, middle, and old age rabbits. In Fig. 3A, reporter ion signals are shown for the light dimethylated precursor of a peptide from fatty acid binding protein (FABP1). This is a triply charged peptide, [N(dimethyl)SVTELNGDTITNTMTIGDVVFK(TMT^10^) +3H]^3+^ and it has both the N-terminal dimethyl group and the lysine TMT^10^ group additions. This precursor was selected for MS/MS and of the MS/MS fragments, 10 fragments were simultaneously subjected to MS^3^. The resulting reporter ions are shown and correspond to an internal pooled QC mixture, and two rabbits each at the young, middle, and old ages. In Fig. 3B, reporter ion signals from the heavy dimethylated peak of this precursor pair are shown in the example MS^3^ spectrum. The reporter ions include the internal standard QC mixture, and also two additional rabbits each at the young, middle, and old ages. Overall, these spectra show that the relative abundances of this FABP1 peptide are higher at young ages and decline in the middle and old ages.

**Table 1:** Biological and technical variation with and without data normalization.

	Raw Data				Normalized Data			
	CV^a^	Fold Cutoff	CV^b^	Fold Cutoff	CV^a^	Fold Cutoff	CV^b^	Fold Cutoff
**Biological replicates**^c^	0.32		0.19		0.29		0.14	
**Technical replicates**^d^	0.36	1.26	0.29	1.17	0.27	1.23	0.17	1.12
Minimum fold cutoff = 1.12, Maximum fold cutoff = 1.26, Mean fold cutoff = 1.195								

a Mean coefficient of variance (CV) of reporter ion intensity with merged light and heavy dimethylated proteins;

b Mean CV of reporter ion intensity with independent light and heavy dimethylated proteins;

c N = 4;

d N = 3 technical injections.

## 2 Experimental design, materials, and methods

### 2.1 Animal husbandry

Male New Zealand white rabbits (Covance, CA) were housed in the Division of Laboratory Animal Resources at the University of Pittsburgh and protocols were approved by the Institutional Animal Care and Use Committee (IACUC). Only male rabbits were included. Liver tissues were harvested from rabbits with a mean age of 252 days (*N* = 4), 683 days (*N* = 4), and 1350 days old (*N* = 4) rabbits and categorized as young, middle, and old age groups, respectively. Liver tissues were directly processed for protein extraction and stored at −80 °C.

### 2.2 Sample preparation

Liver tissues (∼ 100 mg) were added to 1 ml of phosphate buffer saline (PBS) with 8M urea for homogenization. A Fastprep 24 homogenizer (MP Biomedical, CA) was used to lyse liver tissues at 4.0 m/sec for 20 sec with 300 sec pause on ice. The cycle was repeated. Protein extract was collected by centrifugation of lysate at 12,000 rpm, 4 °C, 15 min. Protein concentration was determined using bicinchoninic acid assay (Thermo Scientific, MA) as recommended by the manufacturer. An internal standard was generated by mixing equimolar amounts of proteins from each individual rabbit. Proteins (100 µg) from each sample and internal standard were reduced with dithiothreitol at 1:40 mol ratio, alkylated with iodoacetamide at 1:80 mol ratio, and quenched with L-cysteine at 1:40 mol ratio prior to the addition of a trypsin: Lys-C mix (1:100) enzyme-to-protein ratio. After 18 hours of digestion, peptides were acidified and desalted using a HLB C18 cartridge. Eluted peptides were dried by vacuum centrifugation.

### 2.3 Labeling and high pH RPLC

Desalted peptides (∼50 µg) were reconstituted in 1% acetic acid at a concentration of 0.25 µg/µL. Formaldehyde / ^13^C ^2^H_2_-formaldehyde (60 mM) and sodium cyanoborohydride or sodium cyanoborodeuteride (24 mM) were added to peptides to label the N-termini with either light or heavy dimethyl labels. After 10 minutes, excess label was quenched with the addition of 16 µL of 1% ammonia. Formic acid (5%) was added and dimethylated peptides were desalted and dried down. Dimethylated peptides were reconstituted in 100 mM triethyl ammonium bicarbonate buffer to label the lysine amines with Tandem Mass Tags (TMT10-plex) according to the manufacturer's instructions. Labeled peptides were then pooled to create a single mixture, desalted, and dried down prior to basic pH reverse phase fractionation.

The single mixture of labeled peptides (350 µg) was reconstituted in 1% acetonitrile solution (pH 10.0) and loaded onto an HLB C18 cartridge. Peptides were eluted with a gradual increase of ACN solution in water (3-80%, pH 10.0) into 12 fractions. All fractions were acidified, dried down, and reconstituted in 0.1% formic acid for LC-MS/MS analysis.

### 2.4 LC – MS/MS^n^ and data analyses

Labeled peptides were loaded onto a 2 cm, 3 µm, 100 Å trap column (Thermo Scientific, MA) at 2 µL/min for 10 min. Separation was performed on a 30 cm, 2.5 µm, 130 Å C18 column for 120 min. The gradient was as follows: 0-75 min, 2-30 % mobile phase B; 75-89 min, 30-60 % B; 89-92 min, 60-90 % B; 90-97 min, 90 % B; 97-99 min, 90-10 % B; 99-120 min, 10 % B, where mobile phases (A and B) represent 0.1 % formic acid in water or acetonitrile, respectively. Peptides were detected on a Fusion Lumos Mass Spectrometer with precursor scan parameters as follows: peptides 350-1600 *m/z* were analyzed at 120,000 resolution in the Orbitrap with 50 ms injection time (IT) and 4.0 e5 AGC target. Peptides with 2-8 charges were included. Targeted mass difference of 7.038 and 8.044 Da (±10 ppm tolerance) for light and heavy dimethylated precursor partners was selected. The top most intense ions within 3 sec (Top speed) were selected and isolated for MS/MS fragmentation. 100 ms IT, 1.0 e4 AGC target, 10 ms of 30 % collision energy (CID), and 2 *m/z* isolation window were selected for MS^2^ fragmentation. Fragments ions were detected in the linear ion trap (LIT), followed by an additional fragmentation scan of the ten most intense fragment ions from MS^2^ (synchronous precursor selection) using higher energy collision dissociation (HCD-MS^3^) at a scan range 100-400 *m/z*, 150 ms IT, 5.0e5 AGC target, 55 % collision energy at 60,000 resolution and with 2 *m/z* isolation window. TMT reporter ions were detected in the Orbitrap.

Raw files were searched against the rabbit UniProtKB redundant database (08/19/2018, 21,177 sequences) in Proteome Discoverer software version. 2.2 (Thermo Scientific). A maximum of two trypsin miscleavages were allowed with a precursor mass tolerance of 10 ppm and fragment mass tolerance of 0.6 Da. Light or heavy dimethyl +28.031 or 36.028 Da at N-termini of peptides and carbamidomethylation +57.021 Da (Cys) were selected as static modifications. TMT10-plex +229.163 Da (Lys) and oxidation +15.995 Da (Met) were selected as dynamic modifications. Decoy database search was employed to generate high (p<0.01) and medium (p<0.05) confidence peptide lists. Only rank 1 peptides with peptide deviation of ± 10 ppm were considered while TMT reporter ions (i.e., *m/z* 126 – 129) were identified with ± 30 ppm mass tolerance.

### 2.5 Data normalization and statistics

Each high pH basic reverse phase fraction was analyzed with three technical injections. Later, the reporter ion intensities corresponding to protein groups were summed across 12 fractions while the reporter ion intensities of protein groups were averaged across technical injections. Protein abundance was normalized with an internal reference scaling (IRS) method [3]. Briefly, we summed the protein intensities for each TMT channel and generated a channel-specific scaling factor (SF). The abundance of each individual protein was normalized by the SF of that TMT channel within each technical injection. To normalize the protein abundance across technical triplicates, a protein-specific SF was generated by dividing the geometric mean of internal standard (IS) by the intensity of each protein in the IS channel. A list of statistically-significant proteins was generated through a student's t-test. Proteins with 95% confidence interval and with a fold-change cutoff value of 1.23 were considered significant (p < 0.05).
